# Transcatheter Arterial Infusion of Autologous CD133^+^ Cells for Diabetic Peripheral Artery Disease

**DOI:** 10.1155/2016/6925357

**Published:** 2016-02-14

**Authors:** Xiaoping Zhang, Weishuai Lian, Wensheng Lou, Shilong Han, Chenhui Lu, Keqiang Zuo, Haobo Su, Jichong Xu, Chuanwu Cao, Tao Tang, Zhongzhi Jia, Tao Jin, Georges Uzan, Jianping Gu, Maoquan Li

**Affiliations:** ^1^Department of Interventional and Vascular Surgery, Shanghai Tenth People's Hospital, Tongji University, No. 301 Middle Yan Chang Road, Shanghai 200072, China; ^2^Institution of Interventional and Vascular Surgery, Tongji University, No. 301 Middle Yan Chang Road, Shanghai 200072, China; ^3^Department of Interventional Radiology, Nanjing First Hospital, No. 68 Changle Road, Nanjing, Jiangsu 210001, China; ^4^Unité de Recherche INSERM 602, 94807 Villejuif Cedex, France

## Abstract

Microvascular lesion in diabetic peripheral arterial disease (PAD) still cannot be resolved by current surgical and interventional technique. Endothelial cells have the therapeutic potential to cure microvascular lesion. To evaluate the efficacy and immune-regulatory impact of intra-arterial infusion of autologous CD133^+^ cells, we recruited 53 patients with diabetic PAD (27 of CD133^+^ group and 26 of control group). CD133^+^ cells enriched from patients' PB-MNCs were reinfused intra-arterially. The ulcer healing followed up till 18 months was 100% (3/3) in CD133^+^ group and 60% (3/5) in control group. The amputation rate was 0 (0/27) in CD133^+^ group and 11.54% (3/26) in control group. Compared with the control group, TcPO_2_ and ABI showed obvious improvement at 18 months and significant increasing VEGF and decreasing IL-6 level in the CD133^+^ group within 4 weeks. A reducing trend of proangiogenesis and anti-inflammatory regulation function at 4 weeks after the cells infusion was also found. These results indicated that autologous CD133^+^ cell treatment can effectively improve the perfusion of morbid limb and exert proangiogenesis and anti-inflammatory immune-regulatory impacts by paracrine on tissue microenvironment. The CD133^+^ progenitor cell therapy may be repeated at a fixed interval according to cell life span and immune-regulatory function.

## 1. Backgrounds

Angiopathy and neuropathy are commonly recognized as the basic pathological changes of diabetes, leading to the development of foot complications in diabetic patients [1]. The treatment of purely diabetic neuropathy includes neuronutrition treatment, restricting weight bearing, and foot care therapy, but with limited effects [2]. Ischemia caused by angiopathy is another important factor preventing healing. One of the main features of the angiopathy in patients with diabetic peripheral artery disease (PAD) is that it usually involves both great and small blood vessels.

PAD in diabetics is often multisegmental, typically infrapopliteal, and poorly compensated [3]. With the advancement of surgical skill and the merging of newly developed endovascular instruments, a large number of the aortic-iliac, femoral-popliteal, and infrapopliteal artery lesions can be treated to reestablish sufficient blood supply of the lower limb at macrovascular level; however, there are 20–30% of patients not considered candidates, resulting in amputation as the only option [4, 5]. Moreover, the microvascular lesion in diabetic PAD still cannot be resolved by the current surgical and interventional technique [6].

Endothelial progenitor cells (EPCs) have the capacity of differentiation to mature toward endothelial cells, angiogenesis, and repairmen of impaired vascular walls [7]; therefore, we assumed that EPCs have the therapeutic potential to cure the microvascular lesion of diabetic PAD [8]. Available evidence suggests that local administration of selected and unselected autologous bone marrow derived cells represents a safe and effective means of inducing therapeutic angiogenesis in patients with critical limb ischemia, though the reported benefits were variable [9–13]. Previous studies have demonstrated that cells that express the CD133^+^ surface marker have been characterized as immature progenitor cells with high proliferative, vasculogenic, and regenerative capacity* in vitro* and* in vivo* [14–16]. Although the majority of the population of CD133^+^ positive cells are CD34^+^ positive, they constitute a population of progenitor cells able to differentiate into mature endothelial cells [15]. Importantly, peripheral CD133^+^ positive progenitor cells contain a subpopulation of CD34^−^/133^+^ cells, which are functionally more potent than CD34^+^/CD133^+^ cells [14]. We hypothesized that that autologous CD133^+^ stem cell transplantation may induce vasculogenesis, improve the perfusion of morbid limb, and restore ambulatory function in patients with PAD.

In this study, after successful endovascular infra-aorta revascularization of the macroblood vessels, we enriched CD133^+^ cells from peripheral blood derived mononuclear cells (MNCs) and reinfuse the cells intra-arterially through a catheter to improve the microvascular angiogenesis, evaluating their efficacy and immune-regulatory impact on diabetic subjects with PAD.

## 2. Methods

### 2.1. Study Design

The study was a prospective, nonrandomized trial. All patients assigned to CD133^+^ cells treatment group (CD133^+^ group) and some patients assigned to control group were enrolled in the Tenth People's Hospital of Tongji University (Shanghai, China) and the ethic committee of Tenth People's Hospital of Tongji University approved the protocol. Some patients assigned to the control group were enrolled in the Nanjing First Hospital (Jiangsu, China). All patients provided written informed consent. All patients enrolled were assigned to CD133^+^ cells treatment group (CD133^+^ group) and control group of their own volition. This study is to evaluate the efficacy and immune-regulatory impact of intra-arterial infusion of autologous CD133^+^ cells on diabetic subjects with PAD.

*Clinical Trial Registration*. This trial is registered with URL: http://www.clinicaltrials.gov/. Unique identifier: NCT02474381.

### 2.2. Patient Enrollment and Grouping

Diabetic PAD patients aged ≥18 years with Rutherford categories 2 to 5 were included to assess the eligibility for this study. All patients, who agreed to participate in the study, could voluntarily choose whether to or not to receive autologous CD133^+^ progenitor cell treatment.

In this study, CD133^+^ cells were used to stimulate angiogenesis and reconstruct efficient microvascular blood supply; therefore, similar hemodynamic status in main branch was essential to meet homogeneity in both groups before patient entry. The candidates, who failed in intraluminal revascularization of infra-aorta (iliac and femoral-popliteal) and 1 infrapopliteal (anterior/posterior tibial, fibular) arteries of the affected limb, would be excluded from the study.

Other exclusion criteria were as follows: ① hemoglobin < 10 mg/dL, ② creatinine clearance < 30 mL/min, ③ previous history of stem/progenitor cell therapy, ④ paralysis because of central neural system disease, ⑤ accidental amputation or bone fracture of target limb because of trauma after entry, ⑥ stop of antiplatelet medication after entry, ⑦ smoking or resmoking after entry, and ⑧ malignant tumor.

### 2.3. Treatment of Infra-Aorta and Infrapopliteal Artery Lesion

Computed tomographic angiography (CTA) was performed to firstly analyze the condition of vascular lesion and then digital subtraction angiography (DSA) was performed to precisely identify the lesions of infra-aorta and the infrapopliteal arteries before treatment.

The treatment of infra-aorta artery lesion was restrictedly performed with intraluminal technique (balloon dilation and/or stent implantation) despite the grade of the lesion according to TASC II classification. The arterial sheath was introduced into the contralateral femoral artery, and then the revascularization of the target limb was accomplished by antegrade approach.

After the previous procedure, the lesions of the infrapopliteal arteries were reevaluated by DSA. By means of balloon dilation, at least one of the anterior/posterior tibial and fibular arteries achieved an obvious direct blood supply to the foot.

The goal of the above procedures is to completely restore the normal main trunk hemodynamic status of the target limb.

### 2.4. Autologous CD133^+^ Cells Collection and Preparation

After successful revascularization of infra-aorta and infrapopliteal procedure, 100 mL peripheral blood was collected through the femoral artery sheath in patients and sent to East China Stem Cell Bank (Zhongyuan Union Cell & Gene Engineering Corporation Ltd. (Zhongyuan Union), Tianjin, China) for CD133^+^ cells sorting and enrichment. Mononuclear cells are separated from the whole blood by density gradient centrifugation with Ficoll separating medium (GE Healthcare (Amersham Biosciences), Uppsala, Sweden), and then CD133^+^ cells are selected using magnetic-activated cell sorting with CliniMACS cell separation system (Miltenyi Biotec, Bergisch Gladbach, Germany). The selected cells were mixed with 50 mL sodium chloride injection, which contains human albumin and heparin sodium in the blood bag, and then sent back to hospital stored in 4°C. All collection and preparing procedures were finished within 6 hours.

The selected cells also need to take a quality test; otherwise the cells would be discarded and the patient would be excluded from the study. Selected CD133^+^ cells were tested for viability, purity, and sterility. The quality standards are as follows: cell number ≥ 1 × 10^7^, no visible precipitate in cell suspension, CD133^+^ purity ≥ 97% CD133^+^ based on flow cytometry, viable cell ≥ 90%, and endotoxin ≤ 2 EU/mL.

### 2.5. Cell Infusion Procedure

A catheter was introduced into the popliteal artery of the target limb at tibial plateau level. The CD133^+^ cells suspension was drawn into a 50 mL syringe and infused through the catheter by an injection pump timing to 30 minutes.

For the control group, 50 mL cell-free sodium chloride injection containing human albumin and heparin sodium was infused through the catheter as placebo.

### 2.6. Medication and Life Style Change

Both groups were asked to receive continuous medication for the diabetes, hyperlipidemia, and hypertension under the advice of specialized physicians. Antiplatelet treatment with 100 mg of enteric-coated aspirin and 75 mg clopidogrel daily, as well as statins administration for stabilizing of the arterial plaque, was also demanded.

Besides these medications, all candidates were restrictedly asked to quit smoking after entry.

### 2.7. Follow-Up and Endpoints

The patients were followed up at 18 months.

The primary endpoints were defined as the aggravation of ulcer (developing new or larger or deeper ulcers) and the amputation (above metatarsal level). The ulcer healing and amputation status were observed monthly.

The change of Rutherford classification, TcPO_2_ of dorsum pedis, and ABI were recorded to evaluate the blood perfusion of the limb at 6 and 18 months as the second endpoints.

As proven, the stem cells promote angiogenesis through stimulation of endothelial cell proliferation, migration, and survival by paracrine of high levels of vascular endothelial growth factor (VEGF) [17]. In addition to the regenerative properties, stem cells have an immune-regulatory capacity and induce immunosuppressive effects in a series of situations [18]. Human stem cells have been found to suppress Interleukin-6 (IL-6) expression in activated macrophages, which plays a key role in inflammatory response in wound healing [14]. Thus, the serum concentrations of VEGF and IL-6 before and at 1, 2, and 4 weeks after the CD133^+^ cells infusion were tested to evaluate the proangiogenesis and immunoregulatory impact of the procedure and its duration.

### 2.8. Statistical Analysis

Baseline characteristics were summarized. Independent* t*-test was used to test for differences between the groups for continuous variables and Pearson Chi-Square test was used for categorical variables. Adverse events were summarized. Pearson Chi-Square test was used to test for differences between groups in percent of subjects with amputations. Cox regression tests were used to test for differences in the distributions of amputation-free time. Changes of blood perfusion status, proangiogenesis (VEGF), and immunoregulatory (IL-6) factor in groups are presented descriptively and calculated with Independent* t*-test.

## 3. Results

### 3.1. Baseline Characteristics of Patients

**Figure 1:**
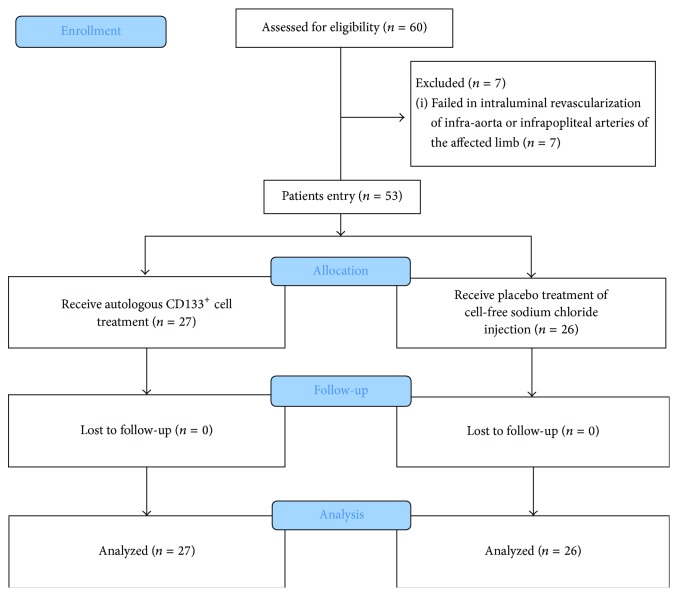
Flow diagram; suggested representation of the flow of participants in a series of diabetic PAD trials.

As the flow diagram (Figure 1) shows, totally 60 subjects were included initially. 30 patients agreed to receive autologous CD133^+^ cells treatment and other 30 patients agreed to participate as control. In CD133^+^ group, 2 patients with TASC II type D lesion failed in the femoral-popliteal revascularization and 1 patient failed in the infrapopliteal revascularization. In control group, 1 patient with TASC II type C lesion and 1 patient with TASC II type D lesion failed in the femoral-popliteal revascularization, and 2 patients failed in the infrapopliteal revascularization. Therefore 53 patients (27 for CD133^+^ group and 26 for control group) were enrolled in the study finally. The baseline characteristics of these patients were described in Table 1. Except for hyperlipidemia, there was no variance of baseline status between the two groups.

### 3.2. Quality of Selected CD133^+^ Cells

From 27 blood samples of CD133^+^ group, the total number of selected cell was 10.29 ± 3.30 (×10^7^, range 5.20–15.20), and the viable cell accounted for 96.88 ± 1.03% (range 95.00–98.70%). No patient showed adverse side effect (skin itching, asthma, blood pressure decrease, and unconsciousness) during their autologous CD133^+^ cells infusion procedure.

### 3.3. Ulcer Healing

Among 53 patients, 8 cases (3 in CD133^+^ group and 5 in control group) were affected with skin ulcer and toe gangrene. In CD133^+^ group, one case had the ulcer on the left heel and one had it on the right big toe, which healed within 1 month; one case had dry gangrene on the right 3 toes (1st, 2nd, and 4th) and healed after 3 months by dropping toes and daily debridement. In control group, 3 cases had toe ulcer healed within 1 month, and 1 case suffered from a new ulcer and 1 case kept unhealed within 18 months. The ulcer healing was 100% (3/3) in CD133^+^ group and 60% (3/5) in control group. There was no significant difference of heal rate between two groups (Pearson Chi-Square, *P* = 0.206).

### 3.4. Amputation

There was no amputation in CD133^+^ group. However, 3 cases in the control group had amputation within 18 months, among which one suffered from new ulceration and being worsening afterwards and got amputation at 5 months, the other one with unhealed ulcer had the amputation at 1 month after enrollment, and the third one had amputation at 2 months, who had never got skin ulcer but suffered from rest pain constantly. The amputation rate was 0 (0/27) in CD133^+^ group and 11.54% (3/26) in control group. There was a trend of fewer and later amputation in CD133^+^ group, but without significant difference in terms of amputation rate (Pearson Chi-Square, *P* = 0.069) or amputation-free time (COX regression, OR 1.125, 95% CI 0.657–1.928, *P* = 0.668) between the two groups.

### 3.5. Rutherford Classification, TcPO_2_ of Dorsum Pedis, and ABI

**Figure 2:**
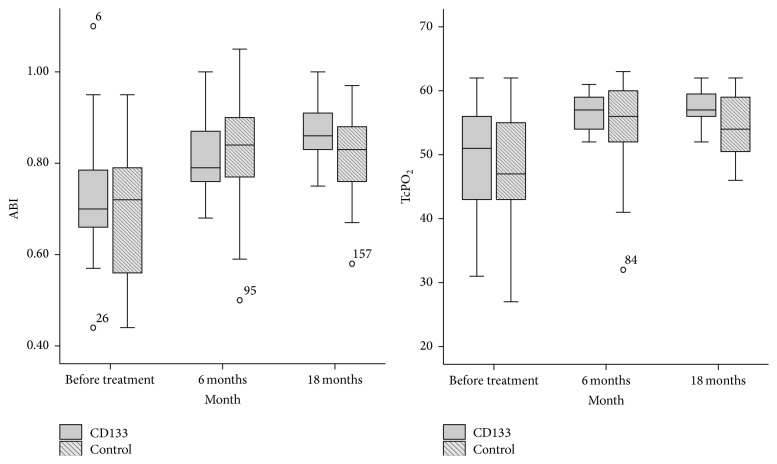
ABI and TcPO_2_ difference before and after treatment. TcPO_2_ and ABI were improved significantly in CD133^+^ group than control at 18 months and did not improve at 1 month, indicating a delayed and persistent perfusion-improving benefit of CD133^+^ progenitor cell treatment. “∘” represents mild outliers in boxplot, and the small numbers beside it represent the case numbers.

The blood perfusion condition of the target limb was assessed by the evaluation of Rutherford classification (Table 2) and the test for TcPO_2_ of dorsum pedis and ABI (Table 3). There was no significant difference observed at 6 months after the procedure; however, the Rutherford classification, TcPO_2_, and ABI showed obvious improvement at 18 months in CD133^+^ group than in control group, which indicated a delayed and persistent perfusion-improving benefit of CD133^+^ cell treatment (Figure 2).

### 3.6. Serum VEGF and IL-6 Concentration

Serum VEGF and IL-6 level in both groups had no difference before the procedure, whereas CD133^+^ group showed significant increasing of VEGF and decreasing IL-6 level compared to the control since the first week (Table 4). There were also trends that the immune-regulatory effect of CD133^+^ cells began to return to basic level at 4 weeks after injected into the patients' limb.

## 4. Discussion

Endothelial cells have the therapeutic potential cure microvascular lesion of diabetic PAD. Many clinical trials have investigated the safety and efficacy of all kinds of EPCs for the treatment of PAD, using cell surface markers-based selected bone marrow or peripheral blood derived MNCs [19]. The most commonly used cell markers is CD34; nevertheless no single marker or combination of markers identifies a pure endothelial progenitor cell population. Researchers have revealed that, with the nature of endothelial cells, CD133 expression is downregulated, whereas CD34 expression is upregulated, and CD34^−^/133^+^ cells subpopulation, a precursor of “classical” CD34^+^/133^+^ EPC, is functionally more potent with respect to homing and angiogenesis [20, 21]. Therefore, we used enriched CD133^+^ cell to initiate microvascular angiogenesis in diabetic PAD patients. Flow cytometry analysis shows that the majority of cells obtained after CD133^+^ positive selection are CD34^+^ positive, the percentage of CD133^+^/34^+^ cells ≥ 98%. This nonrandomized control study firstly reported in the world that CD133^+^ cell provided the proangiogenesis and immune-regulatory effects on diabetic PAD patients.

The results of this study showed the trend of reducing amputation rate, prolonging amputation-free time, and improving ulcer healing of diabetic PAD patients in CD133^+^ group compared to control group. The perfusion status is significantly improved at 18 months in CD133^+^ group, while no significant difference has been found at 6 months, comparing ABI and TcPO_2_ of the affected limb.

The results are consistent with other randomized controlled pilot studies of progenitor cell therapy for critical limb ischemia (CLI). Losordo et al. reported that the autologous CD34^+^ cells slightly reduced the amputation rate than in control group at 6 months (*P* = 0.137) and 12 months (*P* = 0.058) [10]. Fadini et al. analyzed 37 trials of autologous cell therapy for PAD, finding a significant benefit in terms of limb salvage as compared with placebo treatment and the trend toward improving ABI and TcPO_2_ but without significance in cell therapy groups [22]. Theoretically, angiogenesis and vascular repair induced by cell therapy should be a relatively longer-term effect compared to direct endovascular revascularization. Because cells need to be mobilized, adhere to target ischemic tissue, then proliferate, and differentiate to form new vessel structure or vessel wall, all improvement due to cell therapy should not be immediate. Meanwhile, for the limited generation of proliferation and survival duration in diabetic tissue environment of autologous cells, the therapeutic effect should be maintained for restricted period.

Immunoregulation of progenitor/stem cells induced by paracrine manner is another often underestimated factor that contributes to the reperfusion of ischemic area in PAD patients. EPCs are reported to secrete series cytokines and chemokines such as VEGF, HGF, MCP-1, IL-1*β*, IL-6, IL-8, SDF-1, TGF-*β*, TNF-*α*, and M-CSF, inducing critical roles of cell proliferation, migration, tubule formation, homing, and matrix degradation [23].

IL-6 mediates activation, growing, and differentiation of T cells. It stimulates the proliferation and fever response and also induces the production of acute phase proteins with IL-1 [24]. IL-6 can be evaluated as a proinflammatory factor and represented inflammatory level. Researchers have proved that IL-6 levels at the diabetic persons are significantly higher than the healthy ones and this altitude may be a risk factor for the complications like atherosclerosis [25, 26]. In our study, we detect a high level of serum IL-6 concentration in diabetic PAD patients, but after cell treatment, IL-6 concentration in CD133^+^ group remained at lower level than in control group within four weeks (*P* = 0.026, 0.000, and 0.001 at 1, 2, and 4 weeks), indicating a profounder anti-inflammatory regulation effect with the infusion of CD133^+^ cells.

VEGF activates o EPCs and results in new blood vessel formation [27]. The potential of VEGF to induce neovascularization is well established both experimentally and clinically [28]. In our research, we also test the serum VEGF level before and after the treatment. The result showed persistent rising of VEGF in CD133^+^ group more than in control since the first week (79.01 ± 7.75 versus 74.65 ± 6.74 pg/mL, *P* = 0.034) after cell infusion and lasted for at least 4 weeks (82.45 ± 4.74 versus 74.07 ± 8.00 pg/mL, *P* = 0.000, at 2 weeks; 78.61 ± 5.04 versus 72.88 ± 6.64 pg/mL, *P* = 0.001, at 4 weeks), indicating a continuous proangiogenesis effect with the infusion of CD133^+^ cells.

**Figure 3:**
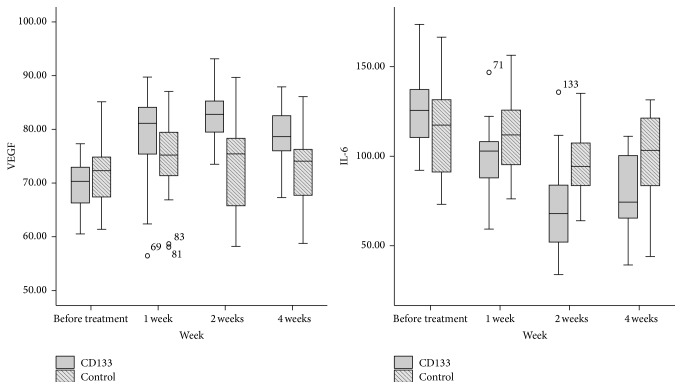
Serum VEGF and IL-6 level change within 4 weeks after treatment. CD133^+^ group had a significant increasing VEGF and decreasing IL-6 level than the control since the first week after treatment, and this significance seemed to become weaker from the 4th week after treatment. “∘” represents mild outliers in boxplot, and the small numbers beside it represent the case numbers.

From the change of VEGF and IL-6, we also firstly found a reducing trend of proangiogenesis and anti-inflammatory regulation function at 4 weeks after the cells infusion (Figure 3). This phenomenon may be caused by the life and proliferation of upper threshold of autologous CD133^+^ cell, which suggest that cell therapy should be repeated at fixed interval according to cell life span.

In conclusion, despite the nonrandomized, no-blinded design and relative small size, results concluded from the study provide initial evidences of the efficacy and safety of autologous CD133^+^ cells treatment for diabetic PAD, as well as the immunoregulatory impact of VEGF and IL-6 paracrine.

## Figures and Tables

**Table 1:** Baseline characteristics of patients.

		CD133^+^	Control	*P* (*t*-test)
Age		71.26 ± 9.12	71.62 ± 9.11	0.887
Gender	Male	12	15	0.901^†^
	Female	12	14	
Type of DM	Type 1	6	9	0.317^†^
	Type 2	21	17	
Hypertension	+	19	21	0.379^†^
	−	8	5	
Hyperlipidemia	+	12	4	0.021^†^
	−	15	22	
Smoking	+	11	10	0.865^†^
	−	16	16	
CAD	+	11	7	0.288^†^
	−	16	19	
Hemoglobin, g/L		120.07 ± 16.77	115.77 ± 15.59	0.338
HbA1c, %		8.26 ± 2.08	8.28 ± 2.07	0.954
Serum creatinine, mL/min		80.60 ± 28.73	79.02 ± 22.44	0.824
Total cholesterol, mmol/L		4.55 ± 1.05	4.58 ± 1.02	0.920
Total triglyceride, mmol/L		2.09 ± 2.36	2.47 ± 2.37	0.560
EMG	+	21	20	0.941^†^
Sensorimotor Polyneuropathy	−	6	6	
TASC II	A	0	2	0.440^†^
	B	11	8	
	C	8	7	
	D	5	3	
No aorta-iliac and femoral-popliteal artery lesions		3	6	
Rutherford	0	0	0	0.320^†^
	1	0	1	
	2	12	5	
	3	8	10	
	4	4	5	
	5	3	5	

DM, diabetes mellitus; CAD, coronary artery disease; EMG, electrical test of the muscles; TASC II, Transatlantic Intersociety Consensus II.

Value were represented by mean ± SD, *t*-test (^†^Pearson Chi-Square).

**Table 2:** Rutherford classification after treatment.

Case number		CD133^+^ case	Control	*P*
6 months	0	1	1	0.114
	1	1	6	
	2	22	13	
	3	3	1	
	4	0	2	
	5	0	1	

18 months	0	1	0	0.004
	1	13	7	
	2	13	6	
	3	0	7	
	4	0	3	
	5	0	0	

Pearson Chi-Square test.

**Table 3:** ABI and TcPO_2_ before and after treatment.

		CD133^+^	Control	*P*
ABI	Before treatment	0.73 ± 0.13	0.70 ± 0.14	0.417
	6 m	0.81 ± 0.08	0.82 ± 0.12	0.733
	18 m	0.86 ± 0.07	0.81 ± 0.09	0.046

TcPO_2_ (mmHg)	Before treatment	48.67 ± 9.14	47.38 ± 9.07	0.610
	6 m	56.37 ± 2.76	54.88 ± 6.75	0.305
	18 m	57.41 ± 2.74	54.35 ± 4.80	0.011

ABI: ankle brachial index; m: month.

**Table 4:** VEGF and IL-6 level before and after treatment.

		CD133^+^	Control	*P*
VEGF (pg/mL)	Before treatment	69.65 ± 4.43	71.68 ± 5.99	0.166
	1 w	79.01 ± 7.75	74.65 ± 6.74	0.034
	2 w	82.45 ± 4.74	74.07 ± 8.00	0.000
	4 w	78.61 ± 5.04	72.88 ± 6.64	0.001

IL-6 (pg/mL)	Before treatment	124.53 ± 19.94	114.24 ± 25.79	0.109
	1 w	98.66 ± 17.85	111.16 ± 21.60	0.026
	2 w	71.00 ± 25.19	96.25 ± 16.98	0.000
	4 w	81.22 ± 20.22	101.16 ± 22.27	0.001

w: week.
